# Supplementary material for: Content-rich biological network constructed by mining PubMed abstracts
Source: BMC Bioinformatics. 2004 Oct 8;5:147. doi: 10.1186/1471-2105-5-147 (PMC528731; doi:10.1186/1471-2105-5-147)
Supplement: Additional File 2 — The original results of the above study (non-essential files are deleted to keep the file size under the limit set by BMC bioinformatics). [file 1471-2105-5-147-S2.bz2 › chilibotAdditionalFile2/dip10/350ID8649415E4594/html/IL3_IL3RA.html]

 


 **IL3** and **IL3RA** 
  
Found 22 abstracts in PubMed, retrieved 10.  
 

 What does Google say? 
 PDF only 
| .edu only 

---

**Interactive relationship** (e.g. stimulation, inhibition, etc)

**Neutral relationship**- Freshly isolated CD123  [ **IL3RA** ]  pre DC showed typical plasmacytoid morphology and when cultured with IL 3  [ **IL3** ]  and CD40L for 72 hr, developed mature DC morphology.  Ref: 12829599 Blood< MedlineTA>Blood,

**Non-interactive relationship** (e.g. studied together, co-existance, homology, etc.)

- According to their morphology, functions, and surface staining they could be separated into two distinct subpopulations mature HLA DRhi, mostly interleukin 3  [ **IL3** ]  R CD123  [ **IL3RA** ]  negative cells, associated with thymocytes, some apoptotic, and expressed myeloid and activation markers but no lymphoid markers.  Ref: 11129651 J Leukoc Biol, 2000
- Interleukin 3  [ **IL3** ]  receptor alpha chain CD123  [ **IL3RA** ]  is widely expressed in hematologic malignancies.  Ref: 11726317 Haematologica, 2001
- The interleukin 3  [ **IL3** ]  IL 3  [ **IL3** ]  receptor CD123  [ **IL3RA** ]  was used to identify basophils in the leukocyte mixture.  Ref: 12587990 Homeopathy, 2003
- DESIGN AND METHODS We analyzed the expression of the interleukin 3  [ **IL3** ]  IL 3  [ **IL3** ]  receptor a chain CD123  [ **IL3RA** ]  in 22 normal samples and in a wide panel of hematologic malignancies using flow cytometry.  Ref: 11726317 Haematologica, 2001
- Two major blood DC populations are found CD11c myeloid DCs, which secrete IL 12, and CD123  [ **IL3RA** ]  IL 3  [ **IL3** ]  receptor DCs lymphoid, which secrete type I interferons in response to viral stimuli.  Ref: 11698285 Blood, 2001
- Like normal basophils, basophils in MDS expressed interleukin 3  [ **IL3** ]  receptor alpha CD123  [ **IL3RA** ] , E NPP3 CD203c, CR1 CD35, CR3 CD11b, CR4 CD11c, membrane co factor protein CD46, decay accelerating factor CD55 and membrane attack complex inhibitory factor CD59, as well as receptors for C3a, C5a CD88, and IgE.  Ref: 11737228 Eur J Clin Invest, 2001
- Although blast cells lacked basophil specific antigens such as CDw17, CD88, and FcepsilonRI, an expression profile of cytokine receptors including CD116 GM CSF receptor, CD117 c kit, and CD123  [ **IL3RA** ]  IL 3  [ **IL3** ]  receptor alpha helped to define the cellular lineage in our case.  Ref: 11999362 Int J Hematol, 2002
- MATERIALS AND METHODS Based on data from 34 patients with hematologic malignancies, doses of CD34 cells and CD34 cell subsets, defined by the expression of HLA DR, CD38, CD117 c kit R, CD123  [ **IL3RA** ]  alpha subunit of IL 3  [ **IL3** ]  R, CD133 AC133, and CD90 Thy 1 antigens, were correlated with the number of short term i.e.  Ref: 11750108 Exp Hematol, 2001
